# Risk Factors and Protective Factors of Internet Addiction in University Students during the Pandemic: Implications for Prevention and Treatment

**DOI:** 10.3390/ijerph20115952

**Published:** 2023-05-25

**Authors:** Daniel T. L. Shek, Wenyu Chai, Kaiji Zhou

**Affiliations:** Department of Applied Social Sciences, The Hong Kong Polytechnic University, Hong Kong

**Keywords:** internet addiction, psychological morbidity, positive psychological attributes, university students, COVID-19 pandemic

## Abstract

While the prevalence rates of Internet addiction (IA) amongst young people during the pandemic are disturbing, few studies have investigated the risk and protective factors of IA in Hong Kong university students under COVID-19. In this study, we examined the relationship between COVID-19-related stress and IA and the role of psychological morbidity and positive psychological attributes in the relationship. In summer 2022, 978 university students completed a survey assessing pandemic-related stress, psychological morbidity, and positive psychological attributes. While psychological morbidity was indexed by depression, post-traumatic stress disorder, and suicidal behavior, positive psychological attributes included life satisfaction, flourishing, adversity beliefs, emotional competence, resilience, and family functioning measures. Results showed that stress and psychological morbidity positively predicted IA, and psychological morbidity mediated the association between stress and IA. Positive psychological attributes negatively predicted stress and IA, and mediated the connection between stress and IA. Positive psychological attributes moderated the mediating effect of psychological morbidity on the relationship between stress and IA. In addition to theoretical contributions, this study contributes to IA prevention and treatment: reducing psychological morbidity and promoting positive psychological attributes are promising strategies to address IA issues in young people.

## 1. Introduction

The COVID-19 pandemic has created many challenges for people in different parts of the world, including university students [1]. Particularly, high prevalence of Internet addiction (IA) in university students was reported during the pandemic [2,3]. This might be attributed to the increased use of the Internet by university students due to campus lockdowns and the shift from face-to-face teaching to online classes [4]. While IA is a common behavioral problem in university students associated with a set of negative outcomes [5,6], limited research has been conducted to explore the risk and protective factors of IA in university students during the pandemic, particularly in a non-Western context such as Hong Kong.

### 1.1. COVID-19 Stress and IA

The COVID-19 pandemic constitutes a significant stressor for university students that might be linked to IA problems. During the pandemic, high levels of COVID-19-related stress were associated with high levels of IA in university students [7,8]. Life stressors under the pandemic also positively predicted IA in university students [9].

### 1.2. Psychological Morbidity and IA

Psychological morbidity is a potential risk factor of IA during the pandemic. Psychological morbidity indexed by depression positively predicted IA in university students during COVID-19 [10,11]; PTSD also positively predicted IA in university students [12,13] and adolescents [14], both before and during the pandemic. In addition, studies reported the predictive role of suicidal behavior in IA [15].

### 1.3. Psychological Morbidity as a Mediator in the Relationship between Pandemic-Related Stress and IA

Research reported the mediating function of depression in the association between perceived stress or stressful life events and mobile phone addiction [16,17]. As COVID-19 related stress predicted depression [18,19] and PTSD [20,21], and depression [22,23] and PTSD [13] predicted IA during the pandemic, we can also hypothesize the mediating function of depression and PTSD in the relationship between pandemic-related stress and IA. Theoretically, the compensatory theory of IA holds that problematic use of the Internet might be a “maladaptive strategy” adopted by university students to deal with both internal and external stressors [24]. As an external stressor, COVID-19 may trigger internal stressors such as mental illness, thus triggering IA [25]. According to the “Interaction-Person-Affect-Cognition-Execution (I-PACE)” model of IA [26], external triggers such as pandemic stress may activate internal triggers, including psychological morbidity, to modify an individual’s cognitive process to engage in addictive behaviors such as IA.

### 1.4. Positive Psychological Attributes and IA

Major positive psychological constructs, such as general satisfaction with life, flourishing, adversity beliefs (positive beliefs), healthy family functioning, emotional competence, and resilience [27,28,29], may play a protective role in IA development in university students. Research reported the protective role of life satisfaction in IA [30,31]. Flourishing was also negatively associated with addictive behaviors, such as substance abuse and Facebook addiction [32,33]. In addition, resilience and emotional competence negatively predicted IA in university students in different studies [34,35]. Positive cultural beliefs about adversity were also a protective factor for addictive behaviors in university students [36]. A negative relationship between healthy family functioning and IA in university students was also reported [37,38].

### 1.5. Positive Psychological Attributes as Mediators and Moderators of the Relationship between Pandemic-Related Stress and IA

Besides acting as a predictive factor, positive psychological attributes may also play a mediating role in the relationship between stress and IA. Life satisfaction mediated the association between pandemic anxiety and social media addiction [39] and the relationship between life stressors and IA [40]. Resilience and emotional intelligence also mediated the association between COVID-19 fear or different stressors and smartphone addiction [41,42,43]. While there is no empirical research examining the mediating role of flourishing, adversity beliefs, and family functioning in the relationship between pandemic stress and IA, their protective nature reported in other studies may imply such an effect [28,29,36]. Since empirical studies suggest the moderating role of positive psychological attributes in the relationship between stressors and psychological morbidity [44,45,46], we propose that positive psychological attributes may moderate the mediating effect of psychological morbidity on the relationship between pandemic stress and IA.

### 1.6. Research Questions and Hypotheses

The previous brief review of the literature shows several research gaps. First, while there are studies examining COVID-19-related stress and IA in university students, there are few studies in Hong Kong. In terms of developmental stages, university students are commonly regarded as people in late adolescence and early adulthood [47,48,49]. Second, there is limited research on the mediating effect of psychological morbidity and positive psychological attributes on the relationship between pandemic stress and IA. Third, there has been no research investigating the moderating role of positive psychological attributes in the mediating effect of psychological morbidity on the relationship between pandemic stress and IA. Fourth, few studies have adopted a comprehensive framework to include multiple risk and protective factors in a single study. To address these research gaps, we generated the following hypotheses in the present paper:

**Hypothesis** **1a:** *What is the relationship between COVID-19 related stress and IA in university students in Hong Kong? Based on past studies [7,8,9], we hypothesized that COVID-19- related stress would positively predict IA in university students in Hong Kong.*

**Hypothesis** **1b:** *Is psychological morbidity related to IA? As existing studies showed a positive association between psychological morbidity (e.g., depression and PTSD) and IA [10,11,12,13], we expected that psychological morbidity would positively predict IA.*

**Hypothesis** **1c:** *Does psychological morbidity mediate the connection between stress related to COVID-19 and IA? With reference to the existing studies [16,17] and related theories on IA [24,26], we expected that psychological morbidity would mediate the predictive effect of stress related to COVID-19 on IA.*

**Hypothesis** **2a:** *Do positive psychological qualities predict IA? Based on the existing studies [31,32,35], we expected that positive psychological attributes would negatively predict IA.*

**Hypothesis** **2b:** *Do positive psychological qualities mediate the relationship between pandemic-related stress and IA? Based on the previous studies [39,40,41], we expected that positive psychological attributes would mediate the predictive effect of pandemic-related stress on IA.*

**Hypothesis** **2c:** *Do positive psychological qualities moderate the mediating effect of psychological morbidity on the linkage between perceived stress and IA? With reference to past studies [44,45,46], we expected that positive psychological qualities would moderate the mediating effect of psychological morbidities on the linkage between COVID-19-related stress and IA.*

**Figure 1:**
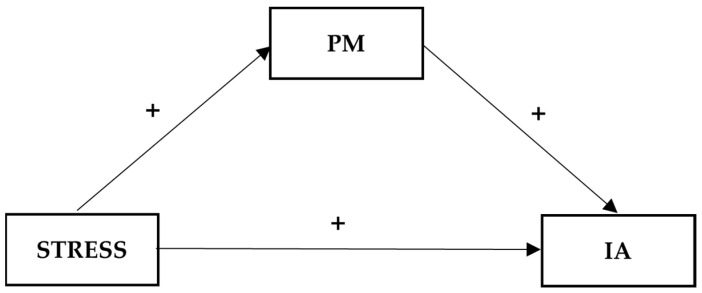
Conceptual model of the mediating effect of psychological morbidity (PM) on the relationship between COVID-19-related stress (STRESS) and Internet addiction (IA).

**Figure 2:**
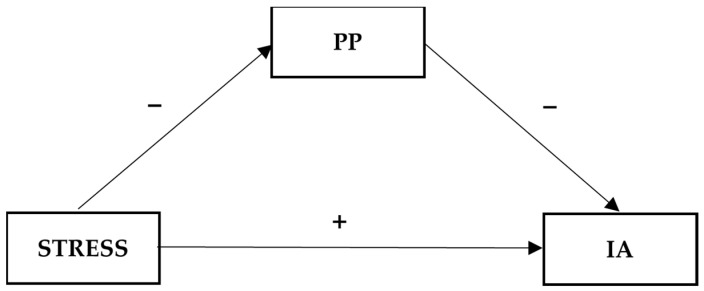
Conceptual model of the mediating effect of positive psychological attributes (PP) on the relationship between COVID-19-related stress (STRESS) and Internet addiction (IA).

**Figure 3:**
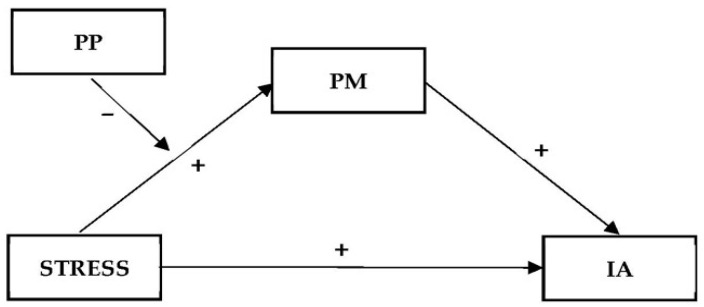
Conceptual model of the moderating effect of positive psychological factors (PP) on the mediating effect of psychological morbidity (PM) on the relationship between COVID-19-related stress (STRESS) and Internet addiction (IA).

The conceptual models for Hypotheses 1c, 2b, and 2c are shown in Figure 1, Figure 2 and Figure 3, respectively. For the mediating and moderated mediation models examined in different hypotheses, we included age, gender, and student status (local vs. international) as covariates since the literature showed that age and gender were important demographic factors associated with IA [10,50] and that international students may have a higher risk of IA [51]. These demographic factors were included as control variables in other related studies in the past [52,53].

## 2. Materials and Methods

### 2.1. Participants and Procedure

In the summer of 2022, undergraduate students from one university in Hong Kong were invited to participate in an online survey via quota sampling with faculty and year of study as two stratifying factors. A total of 978 undergraduate university students participated in the survey and completed the online questionnaire via the Qualtrics XM platform. Approval from the institutional ethics review board and consent from the participants were obtained before data collection. Power analysis using G*power software (Version 3.1.9.4) was conducted for sample size estimation. For multiple linear regression analysis, power was set at 0.95, and the number of independent variables considered in the regression model was six to detect a medium effect size f^2^ = 0.15 (alpha level of 0.05), resulting in a required sample size of 146. We also adopted the Monte Carlo power analysis program for mediation effects developed by Schoemann et al. [54] to check whether our sample size was large enough for mediating analyses. The minimal correlation coefficient among involved variables (STRESS, PM, PP, and IA) was 0.25 based on our observed data (N = 978). A simulation estimated that a sample size of 235 would provide a power of at least 0.8 (lower limit), and a sample size of 285 would provide a power of at least 0.9 (lower limit). This showed that our sample size (N = 978) provided sufficient statistical power to detect the mediating effect.

### 2.2. Measures

#### 2.2.1. Internet Addiction (IA)

Internet addiction was examined through “Young’s 10-item Internet Addiction Test (IAT)” [55]. Shek et al. [56] reported that the measure possessed good psychometric properties. The ten items describe ten addictive symptoms related to Internet use. On each item, the participant answered whether she/he had had the symptom in the past one year using a scale with binary options (“1” = “Yes”, “0” = “No”). The score of IA was gained by summing all item scores.

#### 2.2.2. Stress Related to COVID-19 (STRESS)

The perceived stress related to COVID-19 was measured using four scales. The score of STRESS was the mean score of the four scale scores.

COVID Stress Scale. A revised version of the “COVID Stress Scale” [57] was used in this study to measure perceived stress related to COVID-19. The scale consists of three subscales (each subscale having five items) measuring an individual’s pandemic-related worries in three aspects: “the danger and contamination of COVID-19”, “the socio-economic consequences of COVID-19”, and “the individual’s check behaviors because of concerns about COVID-19”. Each item was evaluated using a five-point scale (“0” = “Not at all” to “4” = “Always”). The scale score was gained by averaging scores of all items. Good reliability of the scale was reported in a previous study (alpha = 0.90) [13].

DASS-Stress. DASS-Stress is one subscale of “Depression Anxiety and Stress Scale (DASS-21)”. It contains seven items assessing perceived stress (in general). DASS-21 was widely used in different studies with good psychometric properties [58,59]. Each item was measured on a scale of four points (“0” = “Not at all” to “3” = “Most of the time”). The score of DASS-Stress was obtained by summing all item scores in this subscale.

Difficulties Encountered during the Pandemic. Difficulties encountered during the pandemic were examined through a self-developed scale with 24 items measuring difficulties and challenges surrounding different life domains during the pandemic [52]. The scale was developed based on findings from student focus group interviews with university students who had very good psychometric properties [52]. Each item was evaluated using a measure with five points (“1” = “Never” to “5” = “Always”). The scale total score was the average of all item scores.

Lockdown/Pandemic Fatigue. The measure was developed to assess lockdown/pandemic fatigue with reference to the Lockdown/Pandemic Fatigue Scale [60] and the Chalder Fatigue Scale [61]. The measure includes seven items corresponding to pandemic fatigue in different aspects, such as physical, mental, and emotional factors. The participants rated to what extent they experienced different pandemic fatigues on a scale with five points (“1” = “Never” to “5” = “Always”). The composite score of fatigue was the mean of all item scores.

#### 2.2.3. Psychological Morbidity (PM)

As depression, post-traumatic stress disorder (PTSD), and suicidal behavior are major types of psychological morbidity [62], they were used in the present study as indicators of psychological morbidity. A composite score was obtained by averaging the scores of the three indicators.

Depression. Depression was examined through the “The Center for Epidemiologic Studies Depression Scale Revised (CESD-R)”. CESD-R (20 items) assesses depression through nine groups of symptoms referring to the “American Psychiatric Association Diagnostic and Statistical Manual (DSM-V)” [63]. The measure was validated in different studies [63,64]. On each item, the student rated how often she/he felt or behaved in the described way using a measure of five points (“0” = “Not at all or less than 1 day” to “4” = “Nearly every day for 2 weeks”). The score of CESD-R was the sum of all item scores.

PTSD. PTSD was assessed through the “Trauma Screening Questionnaire (TSQ)” [65]. With ten items, TSQ asks the participant to indicate whether she/he has had different post-traumatic symptoms “at least twice a week” during the pandemic using a scale with binary options (“0” = “have not experienced”; “1” = “have experienced”). The total score was the sum of all item scores. Previous research has supported the good psychometric properties of TSQ [66].

Suicidal Behavior. Suicidal behavior was evaluated through a measure (3 items) asking the participants’ thoughts, plans, and attempts on suicide in the past one year on a binary scale (“1” = “Yes” and “0” = “No”) [67]. The score of suicidal behavior was the sum of three item scores. The scale showed good reliability in previous research [68].

#### 2.2.4. Positive Psychological Attributes (PP)

The positive psychological attributes were assessed through five indicators described below. A composite score was obtained by averaging scores of the five indicators.

Life satisfaction. “The Satisfaction with Life Scale (SWLS)” was used to examine satisfaction with life [69]. SWLS contains five items evaluating an individual’s general satisfaction with life, with each item rated using a measure of six points (“1” = “Strongly disagree” to “6” = “Strongly agree”). The composite score is the average of all item scores. Good psychometric properties of the scale have been reported [69,70].

Flourishing. Flourishing was examined using the “Flourishing Scale (FS)”. With eight items, FS examines individuals’ psychological well-being through their perceptions of different life aspects, such as life goals, interpersonal relationships, self-esteem, and mental health functioning [71]. Each item was answered on a scale with seven points (“1” = “Strongly disagree” to “7” = “Strongly agree”). The composite score is the mean score of all item scores. Previous research supported the psychometric properties of FS [72].

Beliefs about Adversity. The “Chinese Cultural Beliefs about Adversity (CBA)” scale (nine items) developed by Shek et al. [73] was used to examine beliefs about adversity. For each item, the participant rated to what extent she/he agreed or disagreed with that saying through a scale of six points (“1” = “Strongly disagree” to “6” = “Strongly agree”). The composite score is the average of all item scores. The good psychometric properties of the measure were reported in previous research [74].

Resilience and Emotional Competence. Two subscales in the “Chinese Positive Youth Development Scale (CPYDS)” [75] were used to measure resilience and emotional competence which were found to have good reliability and validity. CPYDS measures the positive development of youth in Chinese societies. It was developed based on the 15 PYD attributes summarized by Catalano et al. [76] and possesses good psychometric properties [75]. Each of the two subscales contains three items rated on a scale of six points (“1” = “Strongly disagree” to “6” = “Strongly agree”). The composite score of resilience and emotional competence was obtained by averaging the scores of the six items.

Family Functioning. Three subscales in the “Chinese Family Assessment Instrument (C-FAI)” [77] were used to examine family functioning, including “Family Communication”, “Family Mutuality”, and “Family Conflict”, which were found to have good reliability and validity. Each item is answered on scale of five points (“1” = “Very unlikely” to “5” = “Very likely”). The score of family functioning was generated by averaging all item scores in which the score of each item in the “Family Conflict” subscale was coded reversely.

### 2.3. Data Analysis

Descriptive statistical and reliability analyses were conducted for all variables. Three composite variables (composite stress (STRESS), psychological morbidity (PM), and positive psychological factors (PP)) were created for further analyses. Correlation analyses were conducted to examine the intercorrelations among the variables under study. The analyses were conducted using SPSS 25. Then, mediation analyses with the BC bootstrap technique (5000 re-samplings) were conducted using PROCESS macro for SPSS (Model 4) to examine the mediating effects of PM and PP on the association between STRESS and IA, respectively. Finally, a moderated mediation model (SPSS PROCESS macro Model 7) was constructed to examine the moderating effect of PP on the mediation effect of PM on the relationship between STRESS and IA.

## 3. Results

The mean age of the participants was 20.69 ± 1.61 years old. There were 336 (34.4%) males and 615 (62.9%) females (with 27 (2.8%) missing); 917 (93.8%) local students and 61 (6.2%) international students (including those from mainland China). There were 418 (42.7%) students in year two, 322 (32.9%) students in year three, and 238 (24.3%) students in year four.

Table 1 shows that the measures used in this study were internally consistent. Cronbach’s α values ranged from 0.65 to 0.96, and inter-item correlations ranged from 0.27 to 0.63.

Table 2 presents the results of the correlation analyses. STRESS variables (COVID stress, DASS-stress, difficulties, fatigue) were positively associated with psychological morbidity indicators (PTSD, depression, and suicidal behavior) and IA (*p*s < 0.01), and the aforementioned variables were all negatively correlated with PP indicators (life satisfaction, adversity beliefs, family functioning, flourishing, resilience, and emotional competence) (*p*s < 0.05). STRESS, psychological morbidity, and IA were positively correlated with each other, and they were negatively correlated with PP (*p*s < 0.001).

Table 3 and Table 4 present results of two mediation analyses, respectively. In Table 3, STRESS positively predicted IA with a high total effect (*B* = 0.98, *p* < 0.001). Psychological morbidity also positively predicted IA (*B* = 0.10, *p* < 0.001). Psychological morbidity partially mediated the association between STRESS and IA (*Estimate* = 0.31, BC 95%CI = [0.16, 0.47], *p* < 0.001) with a significant direct effect (*B* = 0.66, *p* < 0.001). Table 4 shows the mediating effect of PP on the association between STRESS and IA. The total effect (*B* = 0.98, *p* < 0.001) and the direct effect (*B* = 0.89, *p* < 0.001) of STRESS on IA were significant. PP negatively predicted IA (*B* = −0.37, *p* < 0.01) and partially mediated the association between STRESS and IA (*Estimate* = 0.08, BC 95%CI = [0.02, 0.05], *p* < 0.01).

**Figure 4:**
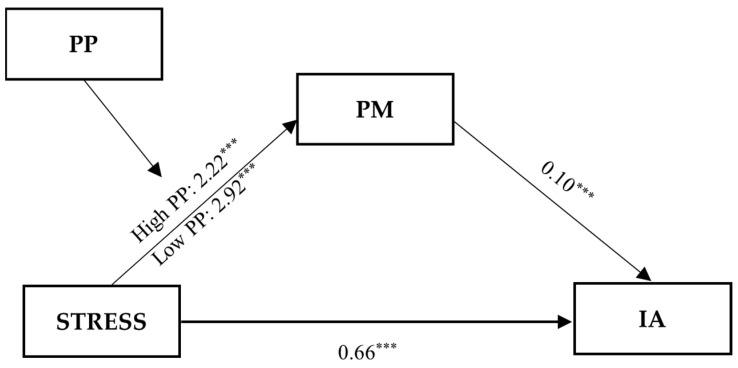
Moderated mediation model predicting Internet addiction (IA). STRESS, composite stress; PP, positive psychological factors; PM, psychological morbidity; IA, internet addiction; *** *p* < 0.001.

**Figure 5:**
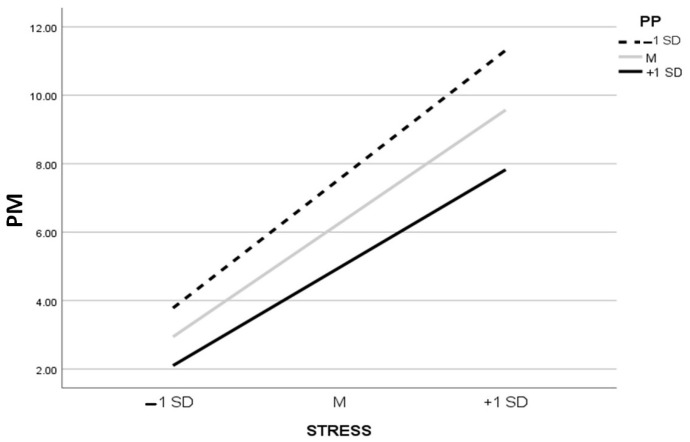
The moderating effect of positive psychological factors (PP) on the relationship between composite stress (STRESS) and psychological morbidity (PM).

Table 5 and Table 6 show results of the moderated mediation analyses (Figure 4 and Figure 5), respectively. STRESS significantly predicted IA through psychological morbidity as a mediator, and PP moderated the mediating effect by mitigating the predictive effect of STRESS on psychological morbidity. The interactive effect between STRESS and PP on psychological morbidity was significant (*B* = −0.55, *p* < 0.001). The predictive effect of STRESS on psychological morbidity was weaker (*B* = 2.22, *p* < 0.001) when PP was high (M + 1 SD), and it was stronger (*B* = 2.92, *p* < 0.001) when PP was low (M–1 SD). Furthermore, the index of moderated mediation was significant (95%CI did not cross 0) (*Index* = −0.06, BC 95%CI = [−0.10, −0.03]), indicating that PP negatively moderated the mediation effect of psychological morbidity. Specifically, the mediation effect of psychological morbidity under high PP was smaller (*Estimate* = 0.23, BC 95%CI = [0.12, 0.34]) than that under low PP (*Estimate* = 0.30, BC 95%CI = [0.16, 0.45]). The contrast between the mediation effects under high PP and low PP was significant (95% CI did not cross 0) (*Contrast* = −0.07, BC 95%CI = [−0.12, −0.03]).

## 4. Discussion

The present study investigated the association between COVID-19 related stress and IA in university students in Hong Kong, with psychological morbidity as a risk factor and positive psychological attributes as protective factors. The study is significant as there is scant literature on this research area in higher education in Hong Kong [13,78]. As IA is a common problem in university students during the pandemic, the study contributes to our understanding of the risk and protective factors of IA and provides direction for effective intervention and prevention programs.

Regarding risk factors, the present study showed that COVID-19 related stress (indexed by COVID stress, general stress, difficulties encountered, and pandemic fatigue) positively predicted IA, which supports Hypothesis 1a. The result is in line with the general literature suggesting a negative association between general stress and IA. For example, higher perceived stress positively predicted higher IA in Indian college students [79]. Perceived stress also positively predicted IA in Malaysian youth [80]. The result was also consistent with the few existing empirical studies on specific COVID-19-related stress and IA. For example, a study on Indonesian college students showed that perceived COVID-19 stress was positively correlated with IA [7]. Based on 6061 medical students in Chinese universities, elevated life stress and uncertainty stress during the pandemic also positively predicted IA [9]. According to compensation theory and the cognitive-behavioral model of IA, students’ engaging in Internet addictive behaviors is a form of “compensation” for perceived stress, which involves a misconception that using the Internet could help them avoid or escape from stress [81,82]. As there is limited research on the predictive effect of COVID-19-related stress on IA in university students, the result of this study contributes to this research area.

Results of this study also showed that psychological morbidity (indexed by depression, PTSD, and suicidal behavior) positively predicted IA in university students, supporting Hypothesis 1b. This is in line with the extant literature. Existing studies showed a positive association between depression and IA in university students [17,18,19]. Depression also predicted a latent profile group of IA in a study on first-year male undergraduate students [23]. In addition, Gavurova et al. [10] found that depression was a significant predictor of IA in Czech and Slovak college students during the pandemic. For PTSD, it positively predicted IA in university students [21] and adolescents [20]. PTSD also predicted IA in university students in Hong Kong during the pandemic [8]. For suicidal behavior, research found that suicidal thoughts and plans positively predicted IA [83]. Taken together, findings of the present study suggest that psychological morbidity is an important risk factor of IA in university students during the pandemic. Theoretically, IA is a problematic behavior developed when individuals use the Internet as a way to temporarily escape from external or internal stressors [24,25]. Zhao et al. [24] also found that escape or avoidance is a central symptom of IA. In short, increased psychological morbidity may constitute a major internal stress which then triggers IA in university students under the pandemic.

In addition, this study showed that psychological morbidity mediated the connection between stress related to COVID-19 and IA, which supports Hypothesis 1c. The findings enrich the existing scientific literature on the underlying mechanisms for the relationship between stress and IA under the pandemic. The relationship between stress and depression has been extensively examined in the scientific literature and the pathway from stress to depression has been theoretically proposed and empirically investigated by scholars in psychology and neuroscience [84,85]. Scientific studies found that stress could lead to brain disturbance which is a central feature of depression [85]. Against the existing literature, it is reasonable to argue that the prolonged stress related to COVID-19 may lead to psychological symptoms which then trigger students’ Internet addictive behaviors as a way to avoid or escape from the psychological symptoms, such as depressive moods.

Regarding the protective factors of IA, positive psychological qualities based on several indicators negatively predicted IA in university students during the pandemic, supporting Hypothesis 2a. The result is consistent with the extant literature. Conceptually, the paradigm of positive psychology highlights the protective function of positive psychological qualities in preventing mental illness and addictive behaviors in youth [27,28]. Particularly, the protective role of important positive constructs, including life satisfaction, flourishing, positive beliefs, resilience, emotional competence, and positive family functioning, were highlighted and were also supported by the empirical literature [31,34,38]. Life satisfaction refers to “the cognitive assessment of one’s life as a whole”, which is not only regarded as an outcome variable but also an important indicator in positive psychology [86] (p. 129). Empirical studies conducted in different contexts suggest the predictive role of life satisfaction in IA [30,31]. Flourishing is defined as an optimal developmental state which comprises “competence, engagement, meaning and purpose, optimism, self-acceptance, supportive relationships” [87] (p. 1). Literature showed that flourishing was negatively associated with addictive behaviors, such as substance abuse and Facebook addiction [32,33]. Resilience and emotional competence, negatively predicted IA in university students in previous studies [34,35]. As far as positive beliefs about adversity are concerned, they are important cultural values about the “nature of adversity such as its causes, consequences and the proper coping behavior” [74] (p. 64). This positive attribute also plays an important role in protecting university students from developing addictive behaviors when experiencing adversities or stress [36,74]. Finally, as an important external asset, positive family functioning was found to be negatively associated with IA in university students [37,38]. Taken as a whole, the findings of this study contribute to the existing theories and literature on positive roles of these psychological attributes. They also echo the recent development that positive psychological attributes are to be emphasized in the higher education sector [71,81,88]. Practically speaking, positive psychological attributes, such as resilience, emotional competence, positive values, and self-awareness, have been incorporated in the graduate attributes of many higher education institutions [73,75].

In addition, results of this study showed that positive psychological attributes mediated the linkage between stress related to COVID-19 and IA in university students, providing support to Hypothesis 2b. The mediating role of positive psychological attributes was also reported in the literature. For example, life satisfaction mediated the association between pandemic anxiety and social media addiction [39]. It also mediated the relationship between life stressors and IA [40]. Resilience was found to be a mediator of the association between COVID-19 fear and smartphone addiction [41], and emotional intelligence mediated the relationship between different stressors and addictive behaviors, such as smoking and smartphone addiction [42,43]. Based on ecological system theory and the positive youth development (PYD) perspective, an individual’s positive functioning and development would be influenced by different environmental factors [89,90]. The increased environmental stress may undermine individuals’ positive psychological attributes [91], hence reducing their protective role in IA.

Furthermore, the present study revealed that positive psychological factors moderated the mediating effect of psychological morbidity on the relationship between pandemic-related stress and IA by moderating the association between pandemic stress and psychological morbidity. While there is richer literature on the predictive effect of positive psychological attributes on IA, the way in which it would protect university students from IA by altering the relationship between stress and IA has not been investigated. This protective role is consistent with the literature suggesting that positive psychological attributes could mitigate the negative effect of stress/stressor on psychological morbidity. For example, life satisfaction moderated the positive effects of academic stress on youth depressive disorder [92]. Emotional intelligence also moderated the positive linkage between peer victimization and IA in primary school students [93]. Based on university students in Hong Kong, a study found that PYD attributes moderated the linkage between need dissatisfaction and depressive disorder during the pandemic [53]. Theoretically, while pandemic-related stress may harm students’ mental health, which increases the risk of IA, students with higher positive psychological attributes, such as resilience, adversity beliefs, and emotional competence, would possess more positive resources to cope with stress and adversity [74] and have better emotional management under stress [35]. This would reduce their risk for developing psychological morbidity, thus reducing their risk for IA. The result suggests a possible pathway through which positive psychological attributes may have protected university students from IA during the pandemic period, which has not been explored in the existing studies.

The present findings have significant implications for assessment, prevention, and treatment of IA. First, as psychological morbidity was identified as a risk factor of IA, it is necessary to understand the psychological morbidity of people suffering from IA in clinical assessment [94,95]. Hence, a more comprehensive and all-round assessment approach should be adopted and “configuring a complete image of the patient’s actual psychopathology” is necessary for assessing IA and devising an effective intervention plan [96] (p. 80). Prevention and treatment of IA might be more effective if the prevention and treatment of related psychological morbidity is involved [97]. As comorbidity is very common in addiction, there is a need to take psychological morbidity of those who have problems of IA into account.

Second, the protective role of positive psychological qualities in IA indicates that the assessment of IA should also consider assessing the positive psychological attributes of the person. In addition, the prevention and intervention of IA should consider promoting the important positive psychological attributes to strengthen protective factors of IA. For example, a study reported the effectiveness of adopting multi-family group therapy (focusing on enhancing parent–child communication and bonding) in reducing IA in adolescents [98]. The project P.A.T.H.S focusing on promoting positive youth development in Hong Kong adolescents was evidenced to be highly effective in reducing IA in adolescents by promoting positive psychological attributes as protective factors in high school and university students [99,100]. In addition, a study on a group-based positive psychology intervention for IA showed effectiveness of the intervention with reduction in IA intensity in the experiment group [101]. However, education on positive psychological attributes is in deficit in Hong Kong [102], and inclusion of assessment of positive psychological attributes in the context of IA is not common.

Despite the insightful nature of the study, several limitations should be noted. First, the research was cross-sectional in nature which cannot give definitive evidence on the causal relationships between the variables. Hence, research based on longitudinal design should be conducted in the future. Second, the study used quota sampling which has the limitation of generalization, although it is commonly adopted in studies under COVID-19 [53]. Third, the participants of the study were recruited from one university in Hong Kong. Further research should include participants from other universities within and outside Hong Kong. Fourth, data were collected at the later stage of Wave 5 of COVID-19 in summer of 2022 during which the perceived stress may not have been as severe as that of the previous waves. Fifth, as students in different study years encounter different situations and tasks, it is interesting to examine the differences in risk and protective factors among students in different study years in future research. Sixth, it is also interesting to examine the relative roles of different risk and protective factors in IA in future research.

## 5. Conclusions

This study addresses the research gap on the risk and protective factors for IA in university students in Hong Kong during the pandemic. It identified psychological morbidity as an important risk factor and positive psychological attributes as important protective factors. The study not only enriches the existing literature on risk and protective factors of IA in university students, but also provides important evidence and direction for better intervention and treatment of IA in university students during the pandemic.

## Figures and Tables

**Table 1:** Descriptive statistics and reliability.

Variable	M	SD	Cronbach’s α	Inter-Item Correlation
STRESS (total)	3.40	1.29	0.95	0.24
COVID stress	1.32	0.76	0.93	0.47
DASS-stress	6.20	4.12	0.88	0.51
Difficulties	3.00	0.60	0.91	0.30
Fatigue	3.06	0.82	0.92	0.61
PM (total)	6.49	5.23	0.95	0.32
PTSD	3.22	2.84	0.82	0.31
Depression	16.04	14.12	0.96	0.54
Suicidal behavior	0.20	0.56	0.65	0.46
PP (total)	3.91	0.64	0.93	0.25
Life satisfaction	3.54	0.98	0.88	0.60
Flourishing	4.62	1.09	0.93	0.63
Beliefs about adversity	3.97	0.68	0.76	0.27
Resilience and emotional competence	4.10	0.82	0.88	0.54
Family functioning	3.33	0.63	0.80	0.32
IA	3.59	2.82	0.81	0.30

Note. STRESS, composite stress; PM, psychological morbidity; PP, positive psychological factors; Fatigue, lockdown, or pandemic fatigue; Difficulties, difficulties encountered under the pandemic; PTSD, posttraumatic stress disorder symptoms; IA, internet addiction.

**Table 2:** Correlations among variables.

Variable	1	2	3	4	5	6	7	8	9	10	11	12	13	14	15
1. STRESS (total)	1														
2. COVID stress	0.56 ***	1													
3. DASS–stress	0.96 ***	0.40 ***	1												
4. Difficulties	0.58 ***	0.41 ***	0.40 ***	1											
5. Fatigue	0.53 ***	0.30 ***	0.33 ***	0.54 ***	1										
6. PM (total)	0.76 ***	0.49 ***	0.75 ***	0.38 ***	0.31 ***	1									
7. PTSD	0.47 ***	0.40 ***	0.40 ***	0.31 ***	0.31 ***	0.56 ***	1								
8. Depression	0.74 ***	0.46 ***	0.74 ***	0.35 ***	0.28 ***	0.98 ***	0.41 ***	1							
9. Suicidal behavior	0.24 ***	0.13 ***	0.24 ***	0.11 ***	0.09 **	0.35 ***	0.25 ***	0.29 ***	1						
10. PP (total)	−0.43 ***	−0.16 ***	−0.45 ***	−0.22 ***	−0.19 ***	−0.52 ***	−0.24 ***	−0.52 ***	−0.23 ***	1					
11. Life satisfaction	−0.26 ***	−0.08 *	−0.24 ***	−0.24 ***	−0.17 ***	−0.28 ***	−0.19 ***	−0.27 ***	−0.16 ***	0.77 ***	1				
12. Flourishing	−0.41 ***	−0.13 ***	−0.42 ***	−0.20 ***	−0.17 ***	−0.49 ***	−0.23 ***	−0.49 ***	−0.21 ***	0.89 ***	0.65 ***	1			
13. BA	−0.31 ***	−0.12 ***	−0.33 ***	−0.10 ***	−0.10 ***	−0.40 ***	−0.16 ***	−0.40 ***	−0.13 ***	0.70 ***	0.37 ***	0.52 ***	1		
14. REC	−0.38 ***	−0.16 ***	−0.42 ***	−0.10 ***	−0.09 ***	−0.47 ***	−0.19 ***	−0.48 ***	−0.18 ***	0.79 ***	0.40 ***	0.66 ***	0.56 ***	1	
15. Family fractioning	−0.28 ***	−0.10 ***	−0.29 ***	−0.15 ***	−0.16 ***	−0.32 ***	−0.13 ***	−0.32 ***	−0.17 ***	0.58 ***	0.31 ***	0.39 ***	0.31 ***	0.36 ***	1
16. IA	0.45 ***	0.31 ***	0.40 ***	0.36 ***	0.28 ***	0.42 ***	0.46 ***	0.37 ***	0.34 ***	−0.25 ***	−0.24 ***	−0.22 ***	−0.14 ***	−0.17 ***	−0.15 ***

Note. STRESS, composite stress; Fatigue, lockdown, or pandemic fatigue; Difficulties, difficulties encountered under the pandemic; PM, psychological morbidity; PTSD, posttraumatic stress disorder symptoms; PP, positive psychological factors; BA, beliefs about adversity; REC, resilience and emotional competence; IA, internet addiction; * *p* < 0.05, ** *p* < 0.01, *** *p* < 0.001.

**Table 3:** Psychological morbidity as a mediator in the relationship between composite stress and Internet addiction.

Path	*B*	*SE*	*t*
Total effect of composite stress (IV) on Internet addiction (DV)	0.98	0.06	15.61 ***
Composite stress (IV) to psychological morbidity (M)	3.06	0.09	35.74 ***
Psychological morbidity (M) to Internet addiction (DV)	0.10	0.02	4.350 ***
Direct effect of composite stress (IV) on Internet addiction (DV)	0.66	0.10	6.98 ***
Mediating effect of psychological morbidity (M)	*Estimate*	BC Bootstrap 95% CI	
		Lower	Upper
	0.31 ***	0.16	0.47

Note. Controlling for gender, student status, age; IV, independent variable; DV, dependent variable; M, mediator; *** *p* < 0.001.

**Table 4:** Positive psychological factors as mediators in the relationship between composite stress and Internet addiction.

Path	*B*	*SE*	*t*
Total effect of composite stress (IV) on Internet addiction (DV)	0.98	0.06	15.61 ***
Composite stress (IV) to positive psychological factors (M)	−0.22	0.01	−15.60 ***
Positive psychological factors (M) to Internet addiction (DV)	−0.37	0.14	−2.62 **
Direct effect of composite stress (IV) on Internet addiction (DV)	0.89	0.07	12.78 ***
Mediating effect of positive psychological factors (M)	*Estimate*	BC Bootstrap 95% CI	
		Lower	Upper
	0.08 **	0.02	0.15

Note. Controlling for gender, student status, age; IV, independent variable; DV, dependent variable; M, mediator; ** *p* < 0.01, *** *p* < 0.001.

**Table 5:** Regression models for moderated mediation analysis.

Predictor Variable	Outcome Variable			
	Psychological Morbidity		Internet Addiction	
	*B*	95% CI	*B*	95% CI
Gender	−0.09	−0.52, 0.34	0.25	−0.08, 0.58
Local	0.13	−0.70, 0.97	−1.06 **	−1.70, −0.42
Age	−0.02	−0.15, 0.10	−0.06	−0.16, 0.03
STRESS	2.57 ***	2.39, 2.75	0.66 ***	0.48, 0.85
PP	−2.02 ***	−2.37, −1.66		
STRESS × PP	−0.55 ***	−0.77, −0.32		
PM			0.10 ***	0.06, 0.15
*F*	266.57 *** (Δ*F* = 22.67 ***)		56.38 ***	
*R* ^2^	0.629 (Δ*R*^2^ = 0.009)		0.230	

Note. Controlling for gender, student status, age; STRESS, composite stress; PP, positive psychological factors; PM, psychological morbidity; Δ*F*, *F* change for interaction term; Δ*R*^2^, *R*^2^ change for interaction term; *** *p* < 0.001; ** *p* < 0.01.

**Table 6:** The mediating effects of psychological morbidity under different levels of positive psychological factors and the index of moderated mediation.

	Level of PP	Effect	SE	BC Bootstrap 95% CI
Mediating effects	Low (M − 1 SD)	0.30	0.07	0.16, 0.44
	Medium (M)	0.26	0.07	0.14, 0.39
	High (M + 1 SD)	0.23	0.06	0.12, 0.34
	Pair	Contrast	SE	BC Bootstrap 95% CI
Pairwise contrast	High-low	−0.07	0.02	−0.12, −0.03
		Index	SE	BC Bootstrap 95% CI
Index of moderated mediation		−0.06	0.02	−0.10, −0.03

Note. Controlling for gender, student status, age; PP, positive psychological factors.

## Data Availability

The data presented in this study are available on request from the corresponding author.
